# Sphingolipid Metabolism and Obesity-Induced Inflammation

**DOI:** 10.3389/fendo.2013.00067

**Published:** 2013-06-04

**Authors:** Se-Chan Kang, Bo-Rahm Kim, Su-Yeon Lee, Tae-Sik Park

**Affiliations:** ^1^Department of Life Science, Gachon University, Seongnam, South Korea

**Keywords:** ceramide, inflammation, obesity, atherosclerosis, cardiomyopathy, fatty liver, diabetes

## Abstract

Obesity is a metabolic disorder developed by overnutrition and a major cause for insulin resistance and cardiovascular events. Since adipose tissue is one of the major sites for the synthesis and secretion of cytokines, enlarged adipose tissue in obese condition alters inflammatory state leading to pathophysiological conditions such as type 2 diabetes and increased cardiovascular risk. A plausible theory for development of metabolic dysregulation is that obesity increases secretion of inflammatory cytokines from adipose tissue and causes a chronic inflammation in the whole body. Additionally accumulation of lipids in non-adipose tissues elevates the cellular levels of bioactive lipids that inhibit the signaling pathways implicated in metabolic regulation together with activated inflammatory response. Recent findings suggest that obesity-induced inflammatory response leads to modulation of sphingolipid metabolism and these bioactive lipids may function as mediators for increased risk of metabolic dysfunction. Importantly, elucidation of mechanism regarding sphingolipid metabolism and inflammatory disease will provide crucial information to development of new therapeutic strategies for the treatment of obesity-induced pathological inflammation.

## Introduction

Obesity is an outcome of overnutrition, less exercise, and sedentary lifestyle. Outcomes of obesity include cardiovascular disease (CVD), diabetes, and hyperlipidemia and contribute to increased mortality and morbidity after myocardial infarction and related complications in diabetic compared with non-diabetic patients (Stone et al., 1989). Even with various current interventions, the occurrence of obesity is increasing and medical expenses associated with curing obesity and its complications are increasing as well (Finkelstein et al., 2009). Deposition of ectopic fat in adipose tissue is associated with increased plasma fatty acids (FA) which are the major contributor to increased lipid contents in non-adipose tissues. Since the adipose tissue is the major place for the pathogenesis of obesity-related metabolic and cardiovascular dysfunction, it has drawn much attention as a target tissue. Obesity worsens tissue functions and contributes to increased risk of development of hypertensions, atherosclerosis, diabetes, and non-alcoholic fatty liver disease (NAFLD) (Flegal et al., 2007). To elucidate the mechanism of pathophysiology of obesity-associated diseases, lipotoxicity and inflammation have been suggested as major contributors to progression of chronic diseases associated with obesity. Among various bioactive lipid metabolites, sphingolipids have been studied due to its implication in development of various chronic metabolic diseases and its bioactive characteristics to modulate cellular signaling pathways (Johns et al., 1998; Shimabukuro et al., 1998; Pettus et al., 2002; Amati et al., 2011). In addition, obesity elevates production of proinflammatory cytokines, chemokines, and coagulation proteins and mediates multiple processes in the body (Hotamisligil et al., 1993, 1995). As a result, inflammation is associated with obese conditions and infiltration of macrophages and T lymphocytes are usually accompanied (Hotamisligil et al., 1993, 1995; Ferrante, 2007). Recent reports demonstrated that sphingolipid metabolism is modulated in obese conditions that alter inflammatory state in adipose tissues and immune cells (Kolak et al., 2007; Holland et al., 2011a). In this review, we will focus on sphingolipid metabolism in the etiology of chronic diseases accompanied with obesity-mediated inflammation. Understanding the role of sphingolipids will provide effective therapeutic targets for obesity-mediated inflammation (Hotamisligil et al., 1993).

## Adipose Tissue Inflammation

**Figure 1:**
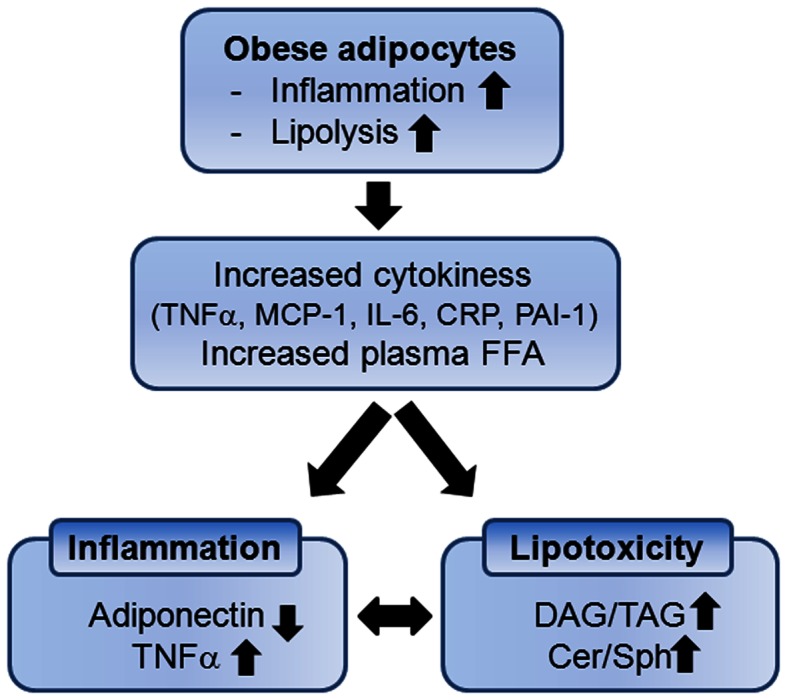
**Enlarged adipocytes by obesity contribute to systemic inflammation and lipotoxicity due to increased cytokines and accumulation of lipid metabolites in non-adipocyte tissues**. DAG, diacylglycerol; TAG, triacylglycerol; Cer, ceramide; Sph, sphingosine.

The adipose tissues has been considered as a lipid storing organ accumulating triglycerides (TAG) in adipocytes in response to overnutrition and releasing these stored lipids during fasting. However, the notion that adipose tissue produces and releases various cytokines, termed “adipokines,” represents this is an active inflammatory organ. The fact that adipose tissue is an active inflammatory organ was initiated from the findings that adipose tissue has increased expression of tumor necrosis factor-α (TNFα) in obese human compared with lean individuals (Hotamisligil et al., 1995). Since this report, it has been reported that adipose tissue from the obese individuals has increased expression and secretion of several proinflammatory cytokines such as tumor necrosis factor-α (TNFα), monocyte chemoattractant protein-1 (MCP-1), interleukin-6 (IL-6), iNOS, C-reactive protein (CRP), and plasminogen activator inhibitor type-1 (PAI-1) (Figure 1) (Mohamed-Ali et al., 1997; Fried et al., 1998; Visser et al., 1999; Perreault and Marette, 2001; Christiansen et al., 2005). Hypertrophied adipocytes induce infiltration of activated macrophages which mediates increased expression and secretion of a variety of proinflammatory cytokines in systemic circulation (Weisberg et al., 2003).

Various adipokines have been suggested as useful biomarkers for CVD and metabolic dysregulation associated with obesity. Depending on the fat contents in the body, the species and the amounts of adipokines secreted from adipose vary. Expression of anti-inflammatory cytokines is upregulated including adiponectin, leptin and IL-10 in response to decreased fat mass (Yang et al., 2001). These adipokines have beneficial effects by regulating body weight due to reduced food intake/energy expenditure and reducing inflammation (Friedman and Leibel, 1992; Zhang et al., 1994; Friedman and Halaas, 1998; Kadowaki et al., 2006). In contrast, expression of proinflammatory cytokines (such as TNFα, MCP-1, and IL-1β) is upregulated with fat mass increase in adipocytes (Jung et al., 2008). Elevation of these cytokines in circulation promotes insulin resistance in peripheral tissues by inhibition of signaling intermediates (Hotamisligil et al., 1995).

Another outcome of obesity is increased lipid accumulation in non-adipose tissues (Kraegen et al., 2001; Unger, 2003). Saturated fat storage capacity of adipose tissue spills free fatty acids (FFAs) in circulation with lipolysis and leads to accumulation of ectopic fat in the tissues not suited for fat storage (Figure 1). Increased FFAs and cytokines activate immune receptors and stress signaling pathways that interfere with insulin signaling in muscle and liver (Holland et al., 2007, 2011a; Hoehn et al., 2008). As non-oxidative pathway of FFAs, intracellular and circulating ceramide are elevated and bioactive sphingolipids such as ceramide, sphingosine, and sphingosine 1-phosphate (S1P) are now known to link overnutrition, inflammation, and metabolic dysregulation.

## Sphingolipid Metabolism in Obesity-Induced Inflammation

Sphingolipid metabolism is highly regulated by a complex network of interconnected pathways not simply by availability of substrate FFAs. Major bioactive sphingolipids includes ceramide, sphingosine, S1P, and ceramide-1-phosphate (C1P) act as signaling molecules regulating various physiological events such as cell proliferation, apoptosis, and inflammation (Futerman and Hannun, 2004; Hannun and Obeid, 2008; Morad and Cabot, 2013). Ceramide is a major molecule in sphingolipid metabolism and a precursor of complex sphingolipids.

**Figure 2:**
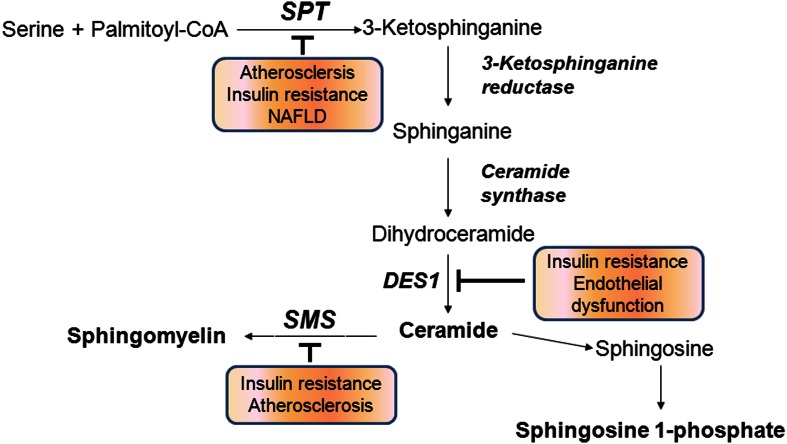
**Sphingolipid biosynthetic pathways**. Inhibition of indicated biosynthetic enzymes is associated with prevention of chronic metabolic diseases. SPT, serine palmitoyltransferase; DES1, dihydroceramide desaturase 1; SMS, sphingomyelin synthase.

*De novo* biosynthesis of ceramide is initiated from condensation of serine and palmitoyl CoA by serine palmitoyltransferase (SPT) followed by a series of reactions involving the enzymes 3-ketosphinganine reductase, ceramide synthase (CerS), dihydroceramide desaturase (DES). Another pathway to produce ceramide is through hydrolysis of sphingomyelin (SM) by acid or neutral sphingomyelinase (SMase) (Hannun and Obeid, 2008) (Figure 2). Ceramide is further deacylated to generate sphingosine by alkaline or acid ceramidase and sphingosine is phosphorylated to produce S1P by sphingosine kinases. Ceramide kinase phosphorylates ceramide to produce C1P (Sugiura et al., 2002). The sphingolipid biosynthesis pathway affects cellular production of at least four known bioactive lipids: ceramide, sphingosine, S1P, and C1P. These signaling lipids are known to alter various physiological events by regulating signaling pathways.

Accumulating evidence suggest that ceramide synthesis can be activated by increased availability of FFAs, proinflammatory cytokines, oxidative stress, and hormones (Memon et al., 1998; Samad et al., 2006; Schilling et al., 2013). All of these conditions represent the obese conditions of adipose tissue and suggest that ceramide metabolism may be altered in the obese. Indeed, ceramide levels were elevated in skeletal muscle, liver, and hypothalamus in obese rodents and human (Adams et al., 2004; Holland et al., 2007; Reyna et al., 2008). Samad et al. (2006) demonstrated that total SM and ceramide levels were reduced in the adipose tissues from the leptin deficient ob/ob mice. In contrast, plasma SM, ceramide, sphingosine, and S1P were elevated in plasma. Since expression of ceramide synthetic genes including SPT, neutral SMase, and acid SMase is upregulated in adipose tissue, this opposite sphingolipid profiles in plasma and adipose tissue suggest that secretion of ceramide from adipose tissues into circulation is increased.

Obesity elevates TNFα expression in adipose tissues (Hotamisligil and Spiegelman, 1994) and ceramide is elevated via hydrolysis of SM by SMases and SPT-mediated *de novo* synthesis. The findings that intraperitoneal administration of TNFα into C57BL/6J mice upregulates acid SMase, neutral SMase, and SPT suggest increased ceramide synthesis in adipose tissue (Samad et al., 2006). To support this report, Holland et al. (2011a) demonstrated that there is an overlap between inflammatory status and ceramide production converging on the Toll-like receptor 4 (TLR4) pathway independent of TNFα signaling. In mutant mice lacking functional TLR4, increased ceramide production by saturated FA or lipopolysaccharides (LPS) was prevented in skeletal muscle and liver. Saturated fat induces ceramide production and inflammatory response in a TLR4-dependent manner. Recently, Schilling et al. (2013) reported that the combination of LPS and palmitate synergistically activates ceramide production via TLR4-dependent and independent signaling respectively. Thus, correlation of ceramide metabolism and inflammatory state has been established by involvement of TLR4 and cytokine-mediated activation of SMase.

## Adiponectin, S1P, and Sphingolipid Metabolism

Obesity increases systemic inflammation state together with production and secretion of proinflammatory cytokines and reduces production of anti-inflammatory cytokines. While TNFα activates proinflammatory pathways and mediates apoptosis, adiponectin inhibits proinflammatory cytokine-mediated pathways and promotes cell proliferation (Ouchi et al., 2000; Kobayashi et al., 2004). Adiponectin is an anti-inflammatory adipokine usually found in circulation (Fang and Sweeney, 2006; Kadowaki et al., 2008). Adiponectin forms three oligomeric forms that can be cleaved by leukocyte elastase to liberate the globular C-terminal fragment which exerts its biological activity (Waki et al., 2005; Wang et al., 2006). Plasma adiponectin levels are generally reduced in the individuals with increasing obesity and diabetes (Liu et al., 2007). Pathophysiological events including diabetes, inflammation, and atherosclerosis are known to be alleviated by adiponectin (Abel et al., 2008; Shetty et al., 2009). These beneficial effects of adiponectin have been attributed to its insulin-sensitizing and insulin-like effects in skeletal muscle and liver. Adiponectin enhances glucose uptake and acts as a stimulator of FA uptake and oxidation via AMP-dependent kinase pathway (AMPK) (Fang and Sweeney, 2006; Kadowaki et al., 2008; Matsuzawa, 2010). Alleviation of lipotoxicity by adiponectin contributes to reduced metabolic dysregulation.

Recent findings by Holland et al. (2011b) demonstrated the linkage between adiponectin and sphingolipid metabolism. Adiponectin receptors contain an inherent ceramidase activity and reduce ceramide. Activity of ceramidase activity is dependent on amount of adiponectin levels and regulated by its biding to the receptors. Adiponectin binding to its two receptors, AdipoR1 and AdipoR2, stimulates ceramidase activity and formation of sphingosine from ceramide degradation is stimulated. Produced sphingosine is phosphorylated by sphingosine kinase to produce S1P, a major bioactive sphingolipid metabolite exerting its anti-apoptotic and anti-diabetic effects. Formed S1P is transported to extracellular environment, binds to the S1P receptors, elevates intracellular calcium, and activates AMPK. Indeed, insulin tolerance is much improved in adiponectin transgenic mice fed a high fat diet when compared to WT mice fed a high fat diet. In opposite to the action of ceramide, S1P has been known to activate Akt and promotes cell proliferation (Morales-Ruiz et al., 2001; Spiegel and Milstien, 2003; Chavez et al., 2005). These findings suggest that inflammatory cytokines are closely linked to modulation of sphingolipid metabolism. S1P and ceramide has opposite roles and regulate the fate of cells for survival or death, “rheostat theory of sphingolipids” in body metabolism.

## Ceramide and Hypothalamic Regulation of Feeding

Elevated FA in circulation is due to obesity-mediated spillover from adipose tissue. Central nervous system regulates appetite and energy homeostasis and hypothalamus controls food intake and its metabolism (Schwartz et al., 2000). Recent reports clearly show that FA in hypothalamus plays an important role in energy balance (Obici et al., 2003; He et al., 2006; Lopez et al., 2006). Especially, malonyl CoA, a precursor of *de novo* FA biosynthesis, draws attentions as a regulator of hypothalamic control (Loftus et al., 2000; Gao and Lane, 2003; Gao et al., 2007). Leptin, an adipokine regulating food intake and body weight, elevates malonyl CoA levels in hypothalamic arcuate nucleus (Arc) and partly causes its anorexigenic effects (Gao et al., 2007). Carnitine palmitoyltransferase-1 (CPT-1) activity, a key enzyme in mitochondrial FA β-oxidation, is inhibited by malonyl CoA and exerts leptin-mediated anorexia (Wolfgang et al., 2007). Wolfgang et al. (2006) have demonstrated hypothalamic CPT-1c, a brain-specific CPT-1 isoform expressed in hypothalamic Arc neuron, is implicated in energy homeostasis. While CPT-1 liver isoform (CPT-1a and -1b) is a predominant form possessing the acyltransferase activity (Obici et al., 2003), CPT-1c has a very weak enzyme activity. Importantly, CPT-1c knockout (KO) animals have reduced food intake and weight gain suggesting a critical role of CPT-1c in energy homeostasis (Wolfgang et al., 2006).

Recently, Gao et al. (2011) have shown that adenoviral overexpression of CPT-1c in hypothalamic Arc increases food intake and upregulates orexigenic neuropeptide Y (NPY) and Bsx, a transcription factor of NPY. Interestingly, CPT-1c overexpression elevated ceramide levels and CPT-1c KO resulted in the opposite, a reduced ceramide levels (Gao et al., 2011). The intra-Arc infusion of C6-ceramide, a cell-penetrating ceramide analog, resulted in blockade of leptin- or cerulenin-mediated anorexigenic effects. In contrast, inhibition of *de novo* ceramide synthesis by myriocin resulted in reduced food intake and body weight. Since CPT-1c and SPT are expressed in ER, palmitoyl CoA might be available via CPT-1c action and supplied for *de novo* ceramide biosynthesis in ER. Another possibility is that CPT-1c acts as a transporter of palmitoyl CoA into ER. Taken together, FA metabolism by CPT-1c exerts its anorexigenic regulation by modulating ceramide synthesis. Although the exact mechanism of CPT-1c/ceramide pathway and regulation of energy homeostasis regarding major Arc neurotransmitters should be studied further, these findings suggested the novel role of ceramide in CNS control of appetite and whole body energy metabolism.

## Involvement of Sphingolipid Metabolism in Development of Chronic Diseases by Obesity

### Insulin resistance

Obesity is closely associated with an increased development of insulin resistance. The concept that inflammation elicited by obese conditions contributes to diabetes was initiated from the findings that adipose-released proinflammatory cytokines inhibit insulin signaling in the adipose, skeletal muscle, and liver (Hotamisligil et al., 1993). Insulin regulates glucose homeostasis by activating glucose uptake by the skeletal muscle and adipose tissue and inhibiting hepatic glucose output. In addition, insulin stimulates FA uptake, TAG biosynthesis, and storage in adipose tissue. Insulin resistance is a pathophysiological process associated with reduced response of target tissues, hyperinsulinemia, and elevated blood glucose levels by increased hepatic glucose efflux. Since obese condition results in increased adipose tissue lipolysis leading to increased plasma FFA, lipotoxicity that accumulation of bioactive lipid intermediates inhibits insulin response has gained credibility for development of insulin resistance. Another possibility is that the obesity activates adipose-derived cytokine production and systemic inflammation and these cytokines disrupt signaling pathways in target tissues. Thus, adipose tissue is a primary location for initiation of insulin resistance and subsequent development of type 2 diabetes.

Free fatty acids are going through oxidative pathway to supply the energy for cell metabolism. Another route for FFAs is sphingolipid biosynthetic pathway. Since FFAs are the substrate and major constituents for sphingolipids, ceramide is elevated in the patients with obesity or diabetes and has a positive correlation with severity of insulin resistance (Haus et al., 2009). Accumulating evidences suggest that sphingolipid metabolism is a converging point linking excess FFAs and inflammation aroused by adipose-derived inflammation, and contributes to progression of insulin resistance. Ceramide and sphingosine inhibit insulin actions and signaling by dephosphorylation and inhibition of AKT and AMPK activity in various cell culture systems (Hajduch et al., 2001; Liu et al., 2004; Summers, 2006). Holland et al. (2007) demonstrated that *in vivo* administration of myriocin, a specific SPT inhibitor, improved glucocorticoid, saturated fat, and obesity-induced insulin resistance by inhibiting *de novo* ceramide synthesis. Heterozygous deficiency of dihydroceramide desaturase (DES1) had improved insulin sensitivity and dexamethasone-induced insulin resistance was prevented (Holland et al., 2007). In cultured cells, the mechanisms of ceramide-mediated inhibition of insulin response have been suggested. It has been demonstrated that ceramide antagonized phosphorylation and activation of AKT and tyrosine phosphorylation of insulin receptor substrate (IRS-1) in 3T3-L1 adipocytes and C2C12 myocytes (Summers et al., 1998; Chavez et al., 2003). Ceramide exerts its inhibitory effects by activating protein phosphatase 2A (PP2A) responsible for dephosphorylation of AKT (Dobrowsky et al., 1993). Additionally, ceramide activates PKCζ and inhibits translocation of AKT to the membrane (Powell et al., 2003, 2004). By modulating AKT activity, ceramide inhibits insulin signaling pathway and ultimately the insulin response is altered.

Since the finding that TNFα is linked to insulin resistance, mechanism of obesity-induced inflammation has been suggested. The activity of c-Jun N terminal kinase (JNK) is increased in the obese mice and the lack of JNK showed improved glucose metabolism (Yuan et al., 2001). To support this finding, inhibition of IKKβ by salicylate was effective in ameliorating inflammation-mediated insulin resistance (Yuan et al., 2001). Since JNK and IKKβ are activated by ceramide, occurrence of decreased insulin resistance can be attributed to tissue ceramide levels (Ruvolo, 2003). Especially, the reports that the absence of functional IKKβ by overexpression of a kinase dead IKKβ decreases ceramide levels in myocytes suggest that IKKβ regulates ceramide biosynthesis (Holland et al., 2011a). Additionally, LPS-mediated NFκB activation in macrophage upregulates transcription of enzymes involved in *de novo* ceramide biosynthesis including Sptlc2 and acid SMase (Chang et al., 2011). Thus, therapeutic intervention that lowers inflammation together with *in vivo* ceramide production would be a good target for obesity-mediated insulin resistance.

### Ceramide and hepatic steatosis

Non-alcoholic fatty liver disease is a component of obesity-mediated complications and defined as excess fat accumulation in the liver (5–10% of liver weight) (Neuschwander-Tetri and Caldwell, 2003). Initial development of NAFLD is the accumulation of TAG in hepatocytes. Benign NAFLD, or hepatic steatosis, at the beginning stage can develop into more malignant conditions exemplified as steatohepatitis and cirrhosis (Farrell and Larter, 2006; Kim and Younossi, 2008). Despite NAFLD is the most common cause of hepatic dysfunction in the United States, its pathogenesis is not completely understood.

Development of NAFLD is associated with overnutrition-mediated obesity. Obesity-induced FFA increase in plasma contributes to approximately 60% of accumulated TAG in the livers of NAFLD patients (Donnelly et al., 2005). In obesity-induced insulin-resistant states, insulin is not able to inhibit the activity of hormone-sensitive lipase in adipose tissues and release FFA into circulation. Additionally, reduced glycerol-3-phosphate levels by insulin resistance prevent reutilization of FFA for TAG synthesis in adipocytes. Therefore, FFA spill from the adipose tissue to circulation is a major cause of NAFLD prevalence. Accordingly, FFA not utilized for TAG synthesis is shunted for ceramide synthesis and ceramide levels are elevated in the adipose tissues from the patients with NAFLD (Kolak et al., 2007). In obese ob/ob mice, hepatic ceramide levels and the degree of steatosis demonstrated a positive correlation (Yetukuri et al., 2007).

Clinically, association of inflammation with NAFLD was confirmed by the fact that patients with NAFLD have elevated levels of TNFα (Jarrar et al., 2008) and expression of TNFα and TNFα receptor are upregulated in the livers of the patients with NAFLD compared to healthy individuals (Feldstein et al., 2004). On the other hand, circulating adiponectin levels were reduced in diet-induced obese (DIO) mice and hepatic expression of adipoR2, a predominant hepatic adiponectin receptor was downregulated (Peng et al., 2009). Additionally, the report that expression of adiponectin is inversely correlated with expression of SMase implies association of inflammation with sphingolipid biosynthesis (Kolak et al., 2007). LPS treatment resulted in a two-fold upregulation of hepatic Sptlc2 mRNA and activity and led to increased hepatic SM and ceramide by twofold and threefold, respectively (Memon et al., 1998). In this study, treatment of IL-1β and TNFα upregulated Sptlc2 mRNA in hepatocytes and indicated that fatty liver induces inflammation-mediated activation of *de novo* biosynthesis.

Implication of SMase, a salvage pathway of ceramide, has been suggested in NAFLD. SMase is regulated by inflammatory stimuli, including TNFα (Dressler et al., 1992; Schutze et al., 1992, 1995; Chatterjee, 1994). Binding of TNFα to p55 TNFα receptor induced SMase transcription (Vandenabeele et al., 1995). Mice deficient in acid SMase and LDL receptors were protected from high fat diet-induced hepatic TAG accumulation (Deevska et al., 2009). Moreover, hyperglycemia and insulin resistance were prevented despite of elevated SM and other sphingolipids (Deevska et al., 2009). In addition, pharmacological inhibition of SMase in palmitic acid-treated hepatocytes reduced TAG significantly (Deevska et al., 2009). These results suggest that SMase-mediated ceramide production is implicated in hepatic steatosis in response to elevated FA. The role of ceramide biosynthesis in development of hepatic steatosis was demonstrated in DIO mice. SPT inhibition by myriocin reduces hepatic TAG in DIO mice (Yang et al., 2009). Reduced hepatic fat accumulation found in myriocin-treated DIO mice implicates downregulation of SOCS-3, a gene involved in development of hepatic steatosis (Ueki et al., 2004). Therefore, regulation of SOCS-3 by ceramide biosynthesis contributes to the pathophysiology of hepatic steatosis. However, whether ceramide contributes to fatty liver directly or via secondary effects such as increased FFA is not clear and need further studies.

### Sphingolipids and atherosclerosis

Obesity-induced inflammation is implicated in increased risk of coronary artery disease. Occurrence of cardiovascular events involves a combined outcome of hyperlipidemia, insulin resistance, hypertension, and heart failure. Recent literature suggests that sphingolipids contribute to pathogenesis of CVD. The fact that sphingolipid metabolism is regulated by inflammatory state suggests that obesity-induced inflammation is the upstream of sphingolipids and risk factors for etiology of various CVD (Holland et al., 2011a). Among them, atherosclerosis is an inflammatory disease characterized by increased production of a wide range of chemokines and cytokines. Early stage of atherogenesis involves the interaction of cholesterol-rich lipoproteins with arterial wall (Ross, 1993). The processes implicated in early atherogenesis include lipoprotein oxidation (Yla-Herttuala et al., 1989; Witztum and Steinberg, 1991), lipoprotein retention and aggregation (Nievelstein et al., 1991; Williams and Tabas, 1995, 1998; Tabas et al., 2007), endothelial alteration, monocyte recruitment, macrophage chemotaxis and foam cell formation, and smooth muscle cell migration and alteration (Ross, 1993). An evidence indicating the importance of SM in atherogenesis is that SM accumulates in atherosclerotic plaques formed in human and animal models (Smith, 1960; Newman et al., 1961; Phillips and Dodge, 1967; Portman and Illingworth, 1976; Hakomori, 1981; Kummerow et al., 2001). LDL extracted from human atherosclerotic lesions has higher SM levels than LDL from plasma (Hoff and Morton, 1985; Guyton and Klemp, 1996; Schissel et al., 1996, 1998). A substantial amount of the SM found in arteries and atherosclerotic lesions appears to arise from SM synthesis in the arterial tissues (Zilversmit et al., 1961; Eisenberg et al., 1969). Plasma SM levels in atherogenic apoE KO mice are fourfold higher than in wild type mice (Jeong et al., 1998) and this may contribute to the increased atherosclerosis (Plump et al., 1992; Zhang et al., 1992). Clinically, Jiang et al. also found that human plasma SM levels and SM/phosphatidylcholine (PC) ratio are independent risk factors for occurrence of coronary heart disease (Jiang et al., 2000; Schlitt et al., 2006).

Park et al. (2004) and Hojjati et al. (2005) have reported that myriocin treatment reduces plasma SM levels and atherosclerosis in apoE KO mice fed normal chow or HFD. While intraperitoneal administration do not alter plasma lipids, myriocin treatment by diet-admix also lower plasma lipid levels in apoE KO mice (Park et al., 2004, 2008b). However, both diet-admix and intraperitoneal administration methods led to reduced atherosclerosis, whereas only oral administration of myriocin lowered plasma cholesterol levels (Park et al., 2004; Hojjati et al., 2005). Oral administration may reduce cholesterol absorption in small intestine (Li et al., 2009). When wild type and apoE KO animals were treated with myriocin, the mice absorbed significantly less cholesterol than controls with no observable pathological changes in the small intestine. Thus, myriocin has direct anti-atherosclerosis vascular effects and also has the potential to function as a plasma lipid-lowering agent. Similar to this study, administration of FTY720, an analog of myriocin, also prevents atherosclerosis in apoE-deficient mice (Liu et al., 2009).

In order to evaluate the role of SM in macrophage, Liu et al. (2009) studied SMS2, SM synthase catalyzing formation of SM from ceramide. In this report, SMS2 KO mouse bone marrow was transplanted into LDL receptor KO *(Ldlr^−/−^)* mice. After 3 months on a Western diet, SMS2 deficiency decreased atherosclerotic lesions in the aortic arch, valve, and the entire aorta, compared with wild type macrophages transplanted into *Ldlr^−/−^* mice. Moreover, the analysis of plaque morphology demonstrated that SMS2 macrophage deficiency resulted in less necrotic core area and more collagen content in atherosclerotic lesions (Liu et al., 2009). Therefore, SMS2 deficiency in the macrophages reduces atherosclerosis in mice.

### Ceramide and cardiomyopathy

Cardiomyopathy is an outcome of various chronic CVDs that is often found in patients with diabetes. Weakening of the heart is sometimes associated with increased heart content of lipids. Diabetic cardiomyopathy contributes to increased morbidity and mortality after myocardial infarction in diabetic patients compared with non-diabetics (Stone et al., 1989). Inflammation is closely associated with development of cardiac events derived from diabetes. Kawamura et al. (2005) inactivates cardiac NFκB by overexpressing a dominant negative NFκB subunit in cardiac-specific TNFα transgenic mice. Although inactivation of NFκB blockage did not improve myocardial inflammation which is represented by inflammatory cell infiltration, it ameliorates cardiac function and mortality. These findings suggest that activation of NFκB is more important than inflammation-mediated immune reaction in cardiomyopathy. To support this observation, TLR4 deficiency attenuates cardiomyopathy in mice (Riad et al., 2008). These findings support that an outcome of inflammation is involved in the etiology of inflammation-induced cardiomyopathy.

Toll-like receptor 4 and NFκB, the mediators of inflammatory response, regulate *de novo* sphingolipid biosynthesis (Chang et al., 2011; Holland et al., 2011a; Schilling et al., 2013). Consistent with these findings, Park et al. (2008a) reported the role of ceramide in a lipotoxic cardiomyopathic mice model. Mice with cardiac overexpression of glycosylphosphatidylinositol membrane-anchored LpL mice (LpL^GPI^) also have increased cardiac ceramide and heart failure markers (Yokoyama et al., 2004). Inhibition of *de novo* ceramide biosynthesis by myriocin or heterozygous deletion of Sptlc1 resulted in decreased expression of some apoptotic markers and ameliorated cardiac contraction in LpL^GPI^ (Park et al., 2008a). In this study, blockage of ceramide biosynthesis appears to modulate mitochondrial substrate oxidation of FA and glucose. A potential mechanism is that decreased ceramide by pharmacological and genetic inhibition of SPT upregulated pyruvate dehydrogenase kinase-4 and decreased the rate of glucose oxidation. However, Lee et al. (2012) reported that ablation of cardiac-specific Sptlc2 (hSptlc2 KO), an essential subunit of SPT, aggravates cardiac function even with reduced ceramide and developed cardiomyopathy. A possible explanation about these inconsistent results is that accumulated FA in hSptlc2 KO hearts due to inhibition of *de novo* ceramide activates ER stress and increases cardiomyocytes apoptosis. Therefore, a single lipid is unlikely to be reduced by inhibition solely and cardiac lipotoxicity is caused by many processes in proper heart function.

### Ceramide and vascular dysfunction

Vascular dysfunction derived from obesity may be mediated by lipotoxic metabolites. A growing body of literature suggests that nitric oxide (NO) is a major modulator to maintain vascular function (Steinberg et al., 2000; Du et al., 2006; Symons et al., 2009). As a ubiquitous signaling molecule, endothelial NO is responsible for regulation of vasodilation (Alderton et al., 2001). Imbalance between production and degradation of NO may lead to occurrence of cardiac events. Obesity mediates increased plasma FFA and increased ceramide contents in various tissues contributing to cardiovascular complications. Especially, ceramide inhibits signaling kinases that phosphorylate endothelial NO synthase (eNOS) at positive regulation site and activates signaling kinases that phosphorylate eNOS at negative regulatory sites (Wu et al., 2007; Chavez and Summers, 2010; Summers, 2010). Recently, Zhang et al. (2012) reported that inhibition of *de novo* ceramide biosynthesis by myriocin ameliorates the blood pressure accrual in mice fed a high fat diet. In this study, prevention of the blood pressure increase by myriocin is endothelium-dependent and via restoration of eNOS phosphorylation at Ser^1177^ (Zhang et al., 2012). Moreover, heterozygous deficiency of dihydroceramide desaturase (des±) partially restored phosphorylation of eNOS, suggesting a major role of ceramide in NO production. A potential mechanism responsible for aggravation of endothelial dysfunction by ceramide is via ceramide-mediated activation of protein phosphatase 2A (PP2A) causing dephosphorylation of eNOS and AKT dissociation. These findings provide the mechanistic link between obesity-induced vascular dysfunction and ceramide.

## Summary

**Figure 3:**
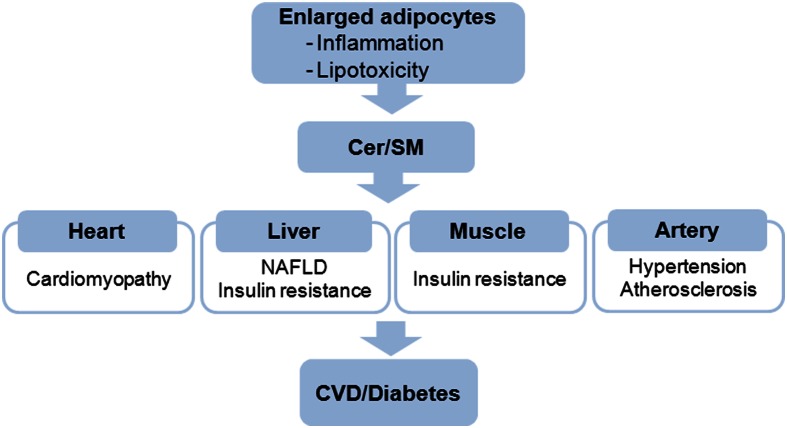
**Elevated ceramide and SM in obese adipocytes elicit the pathophysiological events in various tissues and organs**. CVD, cardiovascular disease.

Obesity manifests in developed countries and contributes to the prevalence of insulin resistance and cardiovascular risk. As a result, obesity-induced inflammation had placed the adipose tissue at the center of inflammation-related pathophysiology. When storage capacity of adipose tissue exceeds its limit for fat deposition, spillage of FFA and adipokines alters inflammatory states in various tissues causing etiology of type 2 diabetes and vascular disease (Figure 3). Due to diverse risk factors for inflammatory disease, it is extremely important to find new approaches for better understanding of the disease. Recent findings suggest that sphingolipids are critical mediators of obesity-mediated inflammation and CVD. SM is implicated as a biochemical modulator of atherosclerosis and ceramide acts as a metabolic switch regulating substrate preference for cardiac energetics and NAFLD (Table 1). Although inflammation and sphingolipid metabolism are closely associated, a number of clinical and experimental issues needs further clarification in future. Moreover, new obesity- and sphingolipid-mediated disease may be found. Thus, modulation of sphingolipid biosynthesis in pathophysiological conditions explained in this review will provide a rationale for therapeutic intervention and present new targets for inflammation-induced chronic diseases.

**Table 1:** **Alteration of tissue ceramide levels and related diseases in animal models of obesity and metabolic syndrome**.

Animal models	Reference	Tissue	Cer/SM changes	Disease related
TLR4 KO mice	Holland et al. (2011a)	Skeletal muscle, liver	Cer ↓	Insulin sensitivity
AdipoR1, AdipoR2 (adenoviral infection into C57BL/6J mice)	Holland et al. (2011b)	Liver	Cer ↓	Insulin sensitivity
CPT-1c KO mice	Gao et al. (2011)	Hypothalamus	Cer ↓	Anorexia
Adipoq KO mice	Holland et al. (2011b)	Heart	Cer ↑	Insulin sensitivity
Des1^±^mice	Holland et al. (2007)	Heart, pancreas, WAT, liver, soleus muscle	Cer ↓	Improved insulin sensitivity
Syrian hamsters + LPS	Memon et al. (1998)	Liver	Cer ↑	Inflammation
Acidic SMase + LDLr^−/−^mice	Deevska et al. (2009)	Liver	Cer ↑	Steatosis
ApoE KO mice	Jeong et al. (1998)	Plasma	SM ↑	Atherosclerosis
SMS2^−/−^, LDLr^−/−^mice	Liu et al. (2009)	Macrophage	Cer ↑SM ↓	Reduced atherosclerosis
DIO mice	Yang et al. (2009)	Plasma, adipose	Cer ↑	Hepatic steatosis, insulin resistance
LpL^GPI^ mice	Park et al. (2008a)	Heart	Cer ↑	Cardiomyopathy
hSptlc2 KO mice	Lee et al. (2012)	Heart	Cer ↓	Cardiomyopathy
ob/ob mice	Samad et al. (2006)	Plasma	Cer ↑, SM ↑	Obesity, diabetes, atherosclerosis
		Adipose	Cer ↓, SM ↓	

*Cer, ceramide; SM, sphingomyelin*.

## Conflict of Interest Statement

The authors declare that the research was conducted in the absence of any commercial or financial relationships that could be construed as a potential conflict of interest.
